# Possible abscopal effect after discontinuation of nivolumab in metastatic renal cell carcinoma

**DOI:** 10.1002/iju5.12195

**Published:** 2020-07-07

**Authors:** Nobuyuki Nakajima, Tatsuo Kano, Kazuya Oda, Takato Uchida, Tatsuya Otaki, Kentaro Nagao, Yuki Shimizu, Masayoshi Kawakami, Hakushi Kim, Masahiro Nitta, Masanori Hasegawa, Yoshiaki Kawamura, Akira Miyajima

**Affiliations:** ^1^ Department of Urology Tokai University Graduate School of Medicine Isehara Kanagawa Japan; ^2^ Department of Urology Tokai University Hachioji Hospital Hachioji Tokyo Japan Japan; ^3^ v Isehara Kyodo Hospital Isehara Kanagawa Japan

**Keywords:** abscopal effect, immune checkpoint inhibitor, programmed cell death‐1, radiotherapy, renal cell carcinoma

## Abstract

**Introduction:**

Renal cell carcinoma has been considered radioresistant. Recently, several studies have reported the efficacy of combination therapy using radiotherapy and immune checkpoint inhibitors.

**Case presentation:**

In 1999, a 56‐year‐old woman underwent left nephrectomy (clear cell carcinoma, pT1bN0M0). Seventeen years postoperatively, recurrence in the left lung hilum was observed. Despite administration of three molecular target drugs, all treatments were terminated due to adverse events. Nivolumab was initiated in December 2016. In August 2017, subcutaneous and lung metastases were observed. Moreover in January 2018, right renal metastasis was noted. After 22 cycles of nivolumab treatment, metastasis in the iliac bone was observed, and the patient was subjected to conventional palliative external beam radiation therapy. Five months after radiotherapy, there was significant reduction in multiple metastases. Here, we reported the case presenting with possible abscopal effect.

**Conclusion:**

Radiotherapy combined with immune checkpoint inhibitors may induce systemic effects against metastatic renal carcinoma.

**Abbreviations & Acronyms**
CT: computed tomography
EBRT: external beam radiation therapy
ICI: immune checkpoint inhibitor
ORR: objective response rate
PD‐1: programmed cell death‐1
RCC: renal cell carcinoma
SRT: stereotactic radiation therapy

**Keynote message**

This report indicates that radiotherapy and anti‐PD‐1 antibody combination therapy is a possible treatment option for metastatic RCC and that nivolumab remains effective for a certain period after its discontinuation.

## Introduction

Inter‐ and intratumor heterogeneity of RCC causes systemic treatment failure and development of resistance.^1^ RCC has been known to be resistant to conventional EBRT. Indeed, an *in vitro* study reported that RCC is the most radioresistant cell line.^2^ The primary application of conventional EBRT is in palliation of metastatic sites and local tumor growth. The phenomenon of tumor regression in distant lesions from the irradiated site is known as the “abscopal effect.” Although the abscopal effect is a rare phenomenon, to the best of our knowledge, there are five case reports on metastatic RCC treated with a combination of ICI and EBRT.^3^, ^4^, ^5^, ^6^, ^7^ Such combination therapies are likely to provide optimal treatment options for advanced RCC.

## Case presentation

**Fig. 1:**
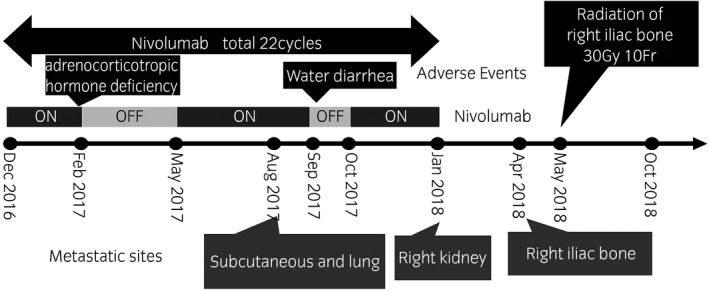
Course of treatment. Nivolumab was initiated in December 2016. In August 2017, subcutaneous and lung metastases were observed. Moreover in January 2018, right renal metastasis was noted. After 22 cycles of nivolumab treatment, metastasis in the iliac bone was observed in April 2018. In May 2018, the patient was subjected to conventional palliative EBRT.

**Fig. 2:**
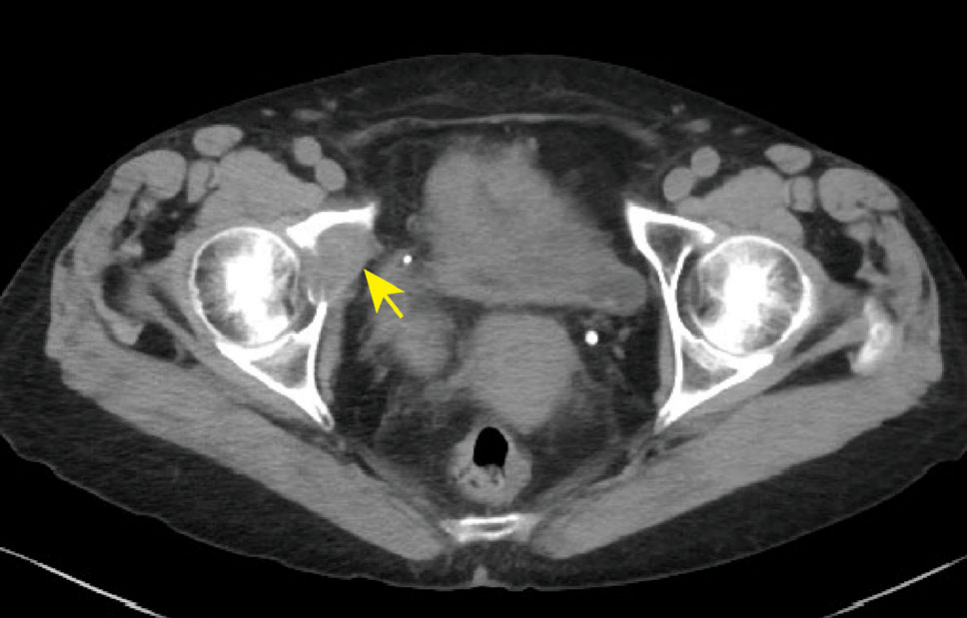
Right iliac bone metastasis. In April 2018, metastasis in the iliac bone was observed by CT scan (arrowhead).

**Fig. 3:**
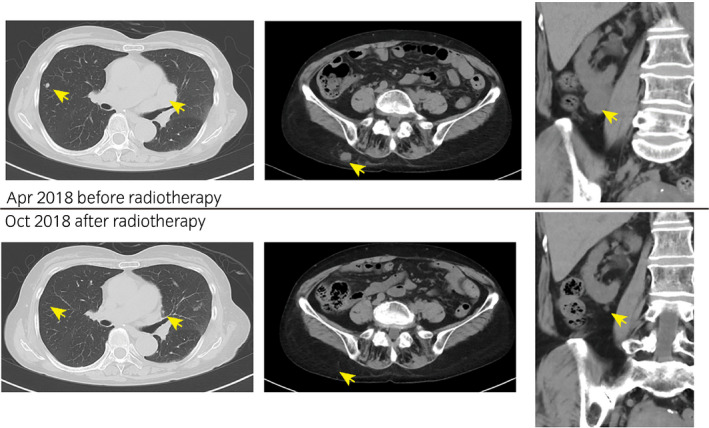
CT scan images before and after radiotherapy. The upper row shows CT scan images of lung metastases (left), subcutaneous metastasis (middle) and right renal metastasis (right) before radiotherapy (April 2018). The lower row shows CT scan images after radiotherapy (October 2018).

In 1999, a 56‐year‐old woman underwent left nephrectomy, which was performed due to identification of RCC with pathological stage pT1bN0M0 clear cell carcinoma. Seventeen years postoperatively, in February 2016, the patient had recurrence in the left lung hilum. The International Metastatic RCC Database Consortium score was 1 (17 years from the diagnosis of the metastasis to initiation of the systemic therapy, hemoglobin level was low, other items were normal), and the International Metastatic RCC Database Consortium status was intermediate risk. Furthermore, pazopanib treatment was initiated in March 2016 and discontinued in May 2016 due to Grade 3 (Common Terminology Criteria for Adverse Events version 4.0) nausea. Consequently, axitinib and everolimus were administered sequentially. However, axitinib and everolimus were discontinued due to Grade 4 chronic renal injury and disease progression, respectively. Finally, nivolumab treatment was initiated in December 2016 and interrupted several times due to adverse events, such as Grade 3 adrenal insufficiency, Grade 3 diarrhea, and Grade 4 chronic renal injury (Fig. 1). In August 2017, subcutaneous and lung metastases were observed. Moreover in January 2018, right renal metastasis was noted. Therefore, nivolumab treatment was discontinued in January 2018 after completion of 22 cycles of administration. Furthermore, in April 2018, CT showed right iliac bone metastasis (Fig. 2). The patient refused to receive further systemic treatment. In May 2018, the right iliac bone was subjected to palliative EBRT (10 fractions of a daily dose of 3 Gy, resulting in a total of 30 Gy). Pain relief was reported after radiotherapy. In October 2018, 5 months after radiotherapy, there was significant reduction in multiple metastases (lung, right kidney, and subcutaneous tissue) (Fig. 3). Nine months after radiotherapy, after 22 cycles of nivolumab administration, no tumor progression was observed, even though no additional treatments were performed. In this case, significant tumor regression of non‐irradiated metastases was observed.

## Discussion

Nivolumab is an anti‐PD‐1 antibody drug. The National Comprehensive Cancer Network Guidelines for Kidney Cancer 2018 recommended nivolumab as an optimal second‐line therapy. The phase III trial of nivolumab (CheckMate 025 trial)^8^ showed prolonged overall survival in patients with metastatic RCC in the second or third line after antiangiogenetic therapy. However, in that trial, ORR and complete response was not as high as 25% and 1%, respectively. In the phase I study of patients treated with SRT and interleukin‐2 combination therapy for metastatic RCC and melanoma, the ORR was 66.7%, and tumor reduction in the non‐irradiated site was observed.^9^ The ORR was higher than that expected with only interleukin‐2 therapy. In another prospective study, patients were treated with SRT in combination with tyrosine kinase inhibitor or ICI. Thirteen of 17 patients (76%) achieved partial response (47%) or complete response (29%).^10^ These results suggest that radiotherapy could enhance the efficacy of systemic therapy. Moreover the efficacy of a combination of radiotherapy and anti‐PD‐1 antibody *in vivo* RCC model has been reported.^11^

The abscopal effect, which was first reported by Mole in 1953, is induced by local radiotherapy and shows the regression of non‐irradiated metastatic lesions that are distant from the irradiated site.^12^ Although it is a rare phenomenon, to the best of our knowledge, there are five case reports on metastatic RCC treated with a combination of ICI and EBRT.^3^, ^4^, ^5^, ^6^, ^7^ The exact mechanisms of abscopal effect have not been elucidated, but some are evident in recent studies.^13^, ^14^ One of the mechanisms is activation of T–cell function. When the tumor cells are irradiated, the damaged tumor cells release damage‐associated molecular patterns, which further activate dendritic cells. The dendritic cells present tumor peptide antigen to naïve T cells. Consequently, the activated T cells migrate to distant metastatic sites and attack tumor cells. Another mechanism involves an increase in immunosensitivity of tumor cells. For example, radiation therapy increases major histocompatibility complex‐class I expression on the surface of irradiated tumor cells.^15^ EBRT, especially SRT, induces the activation of antitumor response, while ICI induces the inhibition of immunosuppression through the PD‐1 antibody. Some studies report that hypofractionated radiotherapy (>5 Gy per fraction) is more effective than conventional fractioned radiotherapy.^16^, ^17^ These studies show that hypofractionated radiotherapy strongly induces antitumor response. In our case, conventional EBRT with 10 fractions of a daily dose of 3 Gy was performed. Although some clinical studies show that radiotherapy followed by ICI or radiotherapy concurrent with ICI is optimal, the sequence and timing of radiotherapy and ICI is still controversial.^16^, ^18^

It is difficult to distinguish the effect of radiotherapy alone and the combined effect of radiotherapy and ICI when they are performed concurrently. In such cases, the abscopal effect may not be noticed even if it occurs. The definition of the abscopal effect does not include the combined use of systemic therapies. In this case, no significant tumor regression was observed during nivolumab therapy, and tumor regression of multiple metastases was observed at 9 months after nivolumab discontinuation. The reduction of non‐irradiated metastases in the lung, right kidney, and subcutaneous tissue after radiotherapy to the right iliac bone appeared to be due to abscopal effect, which may have been enhanced by prior administration of nivolumab. A study reported that the serum half‐life of anti‐PD‐1 antibody was 12–20 days, while a sustained PD‐1 molecule’s mean occupancy of >70% on circulating T cell was >2 months following infusion.^19^ In another study, nivolumab binding to T cell was detected >20 weeks after the last infusion.^20^ In this case, it is likely that the effect was sustained for 4 months after nivolumab discontinuation. Additionally, it was hypothesized that memory‐T‐cell‐mediated immunological memory promoted the abscopal effect after nivolumab discontinuation. In conclusion, nivolumab may have played a certain role in this case.

## Conflict of interest

The authors declare no conflict of interest.
